# Arbuscular Mycorrhizal Fungi Inoculation Promotes Growth and Alleviates *Fusarium tricinctum*-Induced Growth Inhibition in *Quercus variabilis* Seedlings

**DOI:** 10.3390/jof12100776

**Published:** 2026-10-15

**Authors:** Heyu Wang, Yuying Che, Huixin Liu, Xiaohang Yu, Baoxuan Liu, Fengyue Shu, Jing Zhou, Mengmeng Wu

**Affiliations:** 1 School of Life Sciences, Qufu Normal University, Jining 273165, China; 15853230396@163.com (H.W.);; 2 Shandong Key Laboratory of Wetland Ecology and Biodiversity Conservation in the Lower Yellow River, Qufu 273165, China; 3 Rushan City Xujia Town People’s Government, Weihai 264513, China; 4 Shandong Bureau of Hydrology and Water Resources, Yellow River Conservancy Commission, Jinan 250100, China; 5 Shandong Cork Oak Industrial Technology Research Institute Co., Ltd., Jining 272075, China; laucork@pku.org.cn; 6 Shandong Freshwater Fisheries Research Institute, Jinan 250013, China

**Keywords:** plant–microbe interactions, rhizosphere microbiome, nutrient status, fungal challenge, seedling growth

## Abstract

*Quercus variabilis* is a key species for reforestation in East Asia, yet the mechanisms by which arbuscular mycorrhizal fungi (AMF) influence seedling growth and responses to soil-borne fungal challenge remain incompletely understood. This study combined physiological and metagenomic approaches to address this gap. Among seven rhizosphere fungal isolates, *Fusarium tricinctum* (F15) exerted the strongest growth-inhibitory effect on *Q. variabilis* seedlings and was therefore selected for subsequent experiments. Inoculation with the AMF *Rhizophagus intraradices* significantly promoted seedling growth (*p* < 0.05), increasing root length by 4.94% relative to the uninoculated control and increasing ground diameter and aboveground fresh weight by 28.57% and 117.07%, respectively, relative to F15-treated seedlings. AMF inoculation also significantly altered substrate nutrient status (*p* < 0.05), particularly nitrogen, phosphorus, and potassium, and enhanced the activity of antioxidant enzymes, including peroxidase and catalase (*p* < 0.05). Metagenomic analysis revealed that AMF significantly reshaped rhizosphere microbial community composition and bacterial α-diversity (*p* < 0.05), enriching beneficial taxa such as *Mesorhizobium*. Functional profiling showed differences in predicted microbial functional profiles between AMF-inoculated and F15-treated groups. Together, these results indicate that AMF enhances seedling growth and mitigates the negative effects associated with F15 challenge, potentially through altered substrate nutrient dynamics, enhanced antioxidant capacity, and modulation of the rhizosphere microbial community.

## 1. Introduction

The success and sustainability of forest ecosystems depend fundamentally on the health of their foundational species. *Quercus variabilis*, a native oak of East Asia, is ecologically vital and economically valuable for reforestation and timber production [1]. However, its seedlings face significant establishment challenges due to limited soil nutrient availability—particularly phosphorus (P) and nitrogen (N)—and heightened vulnerability to soil-borne pathogens, which collectively constrain growth and survival [2]. Improving nutrient acquisition while enhancing resistance to soil-borne pathogens is therefore important for promoting the establishment and growth of *Q. variabilis* seedlings.

Arbuscular mycorrhizal fungi (AMF) form symbiotic associations with most terrestrial plants and are key to overcoming these constraints [3]. By extending hyphal networks into the soil, AMF vastly expand the plant’s nutrient acquisition capacity, most notably for immobile phosphorus [4], thereby directly enhancing host growth. Improved nutrient acquisition is also closely associated with plant tolerance to biotic stress, because adequate supplies of N, P, and other mineral nutrients support biomass production, photosynthetic activity, and the synthesis of defense-related compounds. Thus, the nutritional benefits provided by AMF may contribute not only to plant growth but also to the capacity of seedlings to withstand pathogen-induced stress. Beyond nutrition, AMF induce systemic resistance in host plants, activating defense pathways that increase tolerance to biotic stresses [5,6]. They enhance disease resistance through multiple mechanisms: competing with pathogens for infection sites, suppressing pathogen proliferation, and stimulating the synthesis of defense compounds [7]. Furthermore, AMF modulate the root microenvironment by influencing phytohormone secretion, enhancing antioxidant enzyme activity, and reshaping the rhizosphere microbial community structure [8].

Increasing evidence has specifically demonstrated interactions between AMF and Fusarium pathogens. AMF inoculation can reduce the incidence or severity of Fusarium wilt and improve plant growth under pathogen challenge, although the magnitude of protection may depend on the AMF species, host genotype, pathogen, and environmental conditions. For example, AMF inoculation has been reported to enhance the suppression of Fusarium wilt in cucumber, accompanied by improved plant biomass and disease control [9,10]. Similarly, inoculation with Rhizophagus irregularis reduced disease severity caused by Fusarium oxysporum in tomato and was associated with enhanced phosphorus acquisition and defense-related responses [11]. These findings indicate that AMF-mediated protection against Fusarium may involve both improved host nutritional status and activation of plant defense mechanisms. However, most previous studies have focused on herbaceous crops, and the interaction between AMF and Fusarium pathogens in woody seedlings remains comparatively less understood.

In addition to direct effects on the host plant, AMF can influence the broader rhizosphere microbiome. The extraradical mycelial network and changes in root physiology and rhizodeposition can modify the availability of carbon and nutrients in the rhizosphere, thereby affecting microbial recruitment and community composition. Rhizosphere bacteria are particularly important because many beneficial bacterial taxa contribute to nutrient cycling, plant growth promotion, and pathogen suppression. Recent work has demonstrated that beneficial rhizobacteria can promote plant growth through nutrient-related functions and suppress soil-borne pathogens through antagonistic and other biocontrol activities [12]. AMF-associated changes in the rhizosphere therefore have the potential to alter microbial interactions and contribute to plant health beyond the direct symbiotic relationship between AMF and the host. In parallel, AMF population density and species diversity are strongly influenced by soil and cropping conditions, highlighting the importance of AMF communities in soil fertility and plant nutrition [13]. However, whether AMF inoculation can improve plant performance under pathogen pressure partly through coordinated changes in rhizosphere bacterial diversity and community structure remains insufficiently understood, particularly in *Q. variabilis* seedlings.

Given this multifaceted role, AMF inoculation holds great potential to improve seedling establishment in forestry. Recent studies underscore its value in enhancing plant growth and disease resistance for reforestation initiatives [13]. Nevertheless, the mechanisms through which AMF simultaneously regulate nutrient status, physiological defense, and rhizosphere microbial communities in *Q. variabilis* seedlings exposed to fungal pathogens remain unclear. An integrated assessment of these processes is therefore necessary to better understand the contribution of AMF to seedling establishment and responses to fungal challenge. This study aimed to investigate the effects of AMF inoculation on the growth and physiological performance of *Q. variabilis* seedlings under F15 challenge, with particular emphasis on associated changes in the rhizosphere microbial community.

## 2. Materials and Methods

### 2.1. Isolation and Molecular Identification of Fungi from the Rhizosphere Soil of Q. variabilis

Rhizosphere soil samples were collected from *Q. variabilis* trees showing poor growth and symptoms of pest or disease damage in Nishan National Forest Park, Qufu, Shandong Province, China. Three rhizosphere soil samples were collected from each of 10 selected trees, resulting in a total of 30 samples. The samples were transported to the laboratory in an insulated container with ice packs and stored at 4 °C until fungal isolation. Fungal colonies were obtained through serial dilution plating on PDA medium at 25 °C [14], with subsequent purification by repeated subculturing. Representative fungal colonies with distinct morphological characteristics were selected for purification. Seven representative fungal isolates, designated F1, F4, F7, F15, BH, NL, and HL, were subsequently selected for ITS amplification and molecular identification. Genomic DNA was amplified using the ITS1 primer (TCC GTA GGT GAA CCTGCG G) and ITS4 primer (TCC TCC GCT TAT TGA TAT GC) [15], followed by sequencing and BLAST (BLAST Version 2.2.28+, http://blast.ncbi.nlm.nih.gov/Blast.cgi, accessed on 6 October 2025) analysis against the NCBI database. Phylogenetic reconstruction was performed using MEGA 6.0 with the Neighbor-Joining (NJ) method [16]. The obtained ITS sequences were deposited in GenBank under accession numbers PZ413545–PZ413551.

### 2.2. Screening of Fungal Isolates for Pathogenicity in Q. variabilis Seedlings

The experiment aimed to evaluate the pathogenicity of seven selected fungal isolates on *Q. variabilis* seedlings. Healthy, 3-month-old *Q. variabilis* seedlings were used as test subjects, cultivated in a substrate composed of peat soil, cork powder, and perlite in a 1:1:2 ratio. The experiment included one control group without inoculation (CK) and seven treatment groups inoculated with the fungal isolates F1, F4, F7, BH, NL, HL, and F15. Prior to the experiment, there were no significant differences among the groups in seedling height, ground diameter, chlorophyll content, or protein content.

The fungal isolates were prepared as spore suspensions with a concentration of 1 × 10^6^ spores/ml [17]. Each seedling was inoculated with 50 mL of spore suspension using the root-drenching method, while the control group received an equal volume of sterile water. Re-inoculation was performed every two weeks, and the changes in the seedlings were closely monitored post-inoculation. Six weeks after the initial inoculation, growth and physiological indicators were assessed.

Seedling height and ground diameter were measured using a ruler and vernier caliper, respectively. Root length was determined by measuring from the root-stem junction to the root tip [18]. Root architecture was quantified using a digital scanning system coupled with WinRHIZO Pro 2007 root analysis software (Regent Instruments Inc., Quebec City, QC, Canada) [19]. Fresh weight, dry weight, and water content of the aboveground and belowground parts of the seedlings were recorded separately [20]. Chlorophyll fluorescence kinetics were assessed after 30-min dark adaptation using a portable fluorometer under 1000 μmol∙m^−2^∙s^−1^ illumination [21]. Photosynthetic parameters—including net photosynthetic rate (Pr), transpiration rate (Tr), stomatal conductance (Gs), and intercellular CO_2_ concentration (Ci)—were measured using a TARGAS-1 portable photosynthesis system (PP Systems, Amesbury, MA, USA) under the following conditions: atmospheric CO_2_ concentration of 477 μmol·mol^−1^, light intensity of 1000 μmol·m^−2^·s^−1^, and temperature of 15–17 °C [22]. Biochemical analyses included chlorophyll quantification via mixed solvent extraction [23] and protein determination by the Coomassie Brilliant Blue method [24].

### 2.3. Effect of AMF Inoculation on Q. variabilis Seedlings Under F15 Challenge

The arbuscular mycorrhizal fungus *Rhizophagus intraradices* was maintained and propagated in the laboratory at the School of Life Sciences, Qufu Normal University, China. The fungal isolate F15, which significantly inhibited the growth of Q. variabilis seedlings, was used in combination with *R. intraradices* to establish four treatments: a non-inoculated control (CK), AMF-only inoculation (AMF), F15-only inoculation (F15), and co-inoculation with AMF and F15 (AMF + F15). *Rhizophagus intraradices* was selected as the AMF inoculum because it is one of the most widespread and dominant AMF species in natural ecosystems [25]. Previous studies have shown that this species frequently dominates AMF communities in relatively heavy soils and has been extensively used in studies investigating plant growth promotion and stress tolerance [26]. Therefore, *R. intraradices* was chosen as the representative AMF species in the present study. Twenty grams of AMF sample was weighed and cultured in a propagation substrate (peat:vermiculite:perlite:sand, 1:1:1:2 *v*/*v*) for 3 months to prepare AMF inoculum. A sterilized substrate (peat:vermiculite:cork powder:perlite, 1:1:1:2 *v*/*v*; autoclaved at 121 °C for 30 min) was loaded into non-woven fabric seedling containers (2/3 full), and a 7 cm cavity was created. Twenty grams of AMF inoculum was evenly applied to the sterilized substrate. Four-month-old *Q. variabilis* seedlings were transplanted into the inoculated substrate and covered with additional sterilized substrate. Plants were watered every 2 days. During the 6-month cultivation period, the seedlings were maintained under a photoperiod of 16 h light/8 h dark, with a light intensity of approximately 200 μmol·m^−2^·s^−1^, an air temperature of approximately 25 °C, and a relative humidity of 60–75%. The substrate water content was maintained at 30–45%. Mycorrhizal colonization was assessed 42 days after transplantation. After six months of cultivation, the effects of AMF inoculation on seedling growth, development, and the rhizosphere microbiome of *Q. variabilis* seedlings were evaluated.

### 2.4. Assessment of AMF Colonization in Q. variabilis Roots

Roots of seedlings were made into 1 cm, which were fixed in FAA fixative for 4 h and heated in 10% KOH at 90 °C for 1 h, followed by alkaline H_2_O_2_ decolorization. Samples were stained with ink-acetic acid solution (5 min boiling), cleared with graded acetic acid rinses, and mounted in lactic acid for microscopic examination. AMF colonization was assessed based on the presence of typical intraradical structures, including hyphae and vesicles. Mycorrhizal colonization rates were quantified from 0 to 100 at 10% intervals using established protocols [27].

$$\mathrm{Infection}\;\mathrm{rate}\%=\frac{\sum (0\times \;\mathrm{segments}+10\times \;\mathrm{segments}+20\times \;\mathrm{segments}+\ldots 100\times \;\mathrm{segments})}{\mathrm{observed}\;\mathrm{root}\;\mathrm{segments}}$$

### 2.5. Substrate Nutrient Determination in Q. variabilis Seedlings

Soil chemical properties were analyzed following the methodology of Lu 2000 [28]. pH measurement utilized 2 g subsamples through a gravimetric approach. Total nitrogen quantification involved sulfuric acid-potassium permanganate digestion with semi-automatic Kjeldahl analysis. Total phosphorus required perchloric-sulfuric acid digestion, while total potassium employed tri-acid digestion with nitric-perchloric-hydrofluoric acids. Available phosphorus extraction used fluoride-hydrochloric acid on 5 g samples, and available potassium was assessed via ammonium acetate extraction combined with flame spectrophotometry. Organic matter determination applied dichromate oxidation to 0.5 g samples. Ammonium nitrogen extraction with potassium chloride followed by the indophenol blue method was implemented for 5 g samples. Alkali-hydrolyzed nitrogen was evaluated through an alkaline diffusion protocol.

For soil physicochemical analyses conducted by external certified laboratories, three independent biological replicates were used due to analytical and cost constraints, whereas five biological replicates were used for in-house measurements.

### 2.6. Determination of Rhizosphere Microorganisms and Microbial Diversity in Q. variabilis Seedlings

Soil genomic DNA was extracted from 0.5 g soil samples using the FastDNA Spin Kit (MP Biomedicals, Solon, OH, USA), and DNA quality was assessed by electrophoresis on 1% agarose gels. Metagenomic sequencing was performed by Shanghai Majorbio Bio-pharm Technology Co., Ltd. (Shanghai, China) using the NovaSeq X Plus platform. The obtained sequences were deposited in the NCBI Sequence Read Archive (SRA) under accession number PRJNA1088856.

Raw sequencing reads were subjected to quality control to remove low-quality reads and reads containing ambiguous bases (N), resulting in high-quality clean reads for subsequent analyses. High-quality reads were assembled into contigs using an appropriate assembly strategy, and the assembled contigs were used for open reading frame (ORF) prediction. ORFs were predicted from contigs using Prodigal v2.6.3 (https://github.com/hyattpd/Prodigal, accessed on 6 October 2025), and genes with nucleotide lengths ≥ 100 bp were retained and translated into amino acid sequences.

Predicted gene sequences from all samples were clustered using CD-HIT (https://github.com/weizhongli/cdhit, accessed on 6 October 2025) [29] to construct a non-redundant gene catalog, with the longest sequence in each cluster selected as the representative sequence. SOAPaligner version 2.21 [30] was used to map high-quality reads from each sample to the non-redundant gene set for gene abundance quantification.

For taxonomic annotation, the non-redundant gene set was aligned against the NCBI NR database using BLASTP (BLAST Version 2.2.28+, http://blast.ncbi.nlm.nih.gov/Blast.cgi, accessed on 6 October 2025) [31]. Taxonomic information was assigned at the domain, kingdom, phylum, class, order, family, genus, and species levels, and the abundance of each taxon was calculated based on the summed abundance of the corresponding genes. Based on the taxonomic abundance profiles, α-diversity was evaluated using the ACE, Shannon, Simpson, Shannon evenness, and Simpson evenness indices. β-diversity was assessed based on Bray–Curtis distances, followed by principal coordinates analysis (PCoA) to visualize differences in microbial community composition among treatments. ANOSIM was performed to statistically evaluate differences in microbial community composition among treatments.

Functional annotation was performed by aligning protein sequences against the KEGG database using BLASTP, followed by annotation using KOBAS 2.0 [32]. The abundance of each functional category was calculated as the sum of gene abundances assigned to KO, Pathway, EC, and Module classifications. Differential pathway analysis among treatments was subsequently performed.

### 2.7. Statistical Analysis

All experimental data were expressed as mean ± standard deviation (SD). One-way analysis of variance (ANOVA) followed by Tukey’s multiple-comparison test was used to evaluate differences among treatments in substrate physicochemical properties, seedling growth and physiological indicators, and bacterial and fungal community α-diversity indices. A *p* value < 0.05 was considered statistically significant [33].

## 3. Results

### 3.1. Molecular Identification and Analysis of Fungal Isolates

The fungal isolates were identified based on ITS sequence analysis. The ITS sequences obtained from isolates F1, F4, F7, F15, BH, NL, and HL ranged from 561 to 704 bp in length and were deposited in the GenBank database under accession numbers PZ413545–PZ413551.

BLAST analysis revealed that isolate F1 shared 99.70% sequence similarity with *Gongronella butleri* based on an ITS sequence of 664 bp, while isolate F7 showed 98.95% similarity with *Gongronella hydei* based on an ITS sequence of 688 bp. Isolate F4 (704 bp) shared 99.43% similarity with *Cunninghamella elegans*, isolate F15 (561 bp) shared 99.80% similarity with *Fusarium tricinctum*, isolate BH (607 bp) shared 99.31% similarity with Aspergillus niger, isolate NL (590 bp) shared 100% similarity with *Penicillium oxalicum*, and isolate HL (579 bp) shared 99.80% similarity with *Talaromyces funiculosus*.

**Figure 1:**
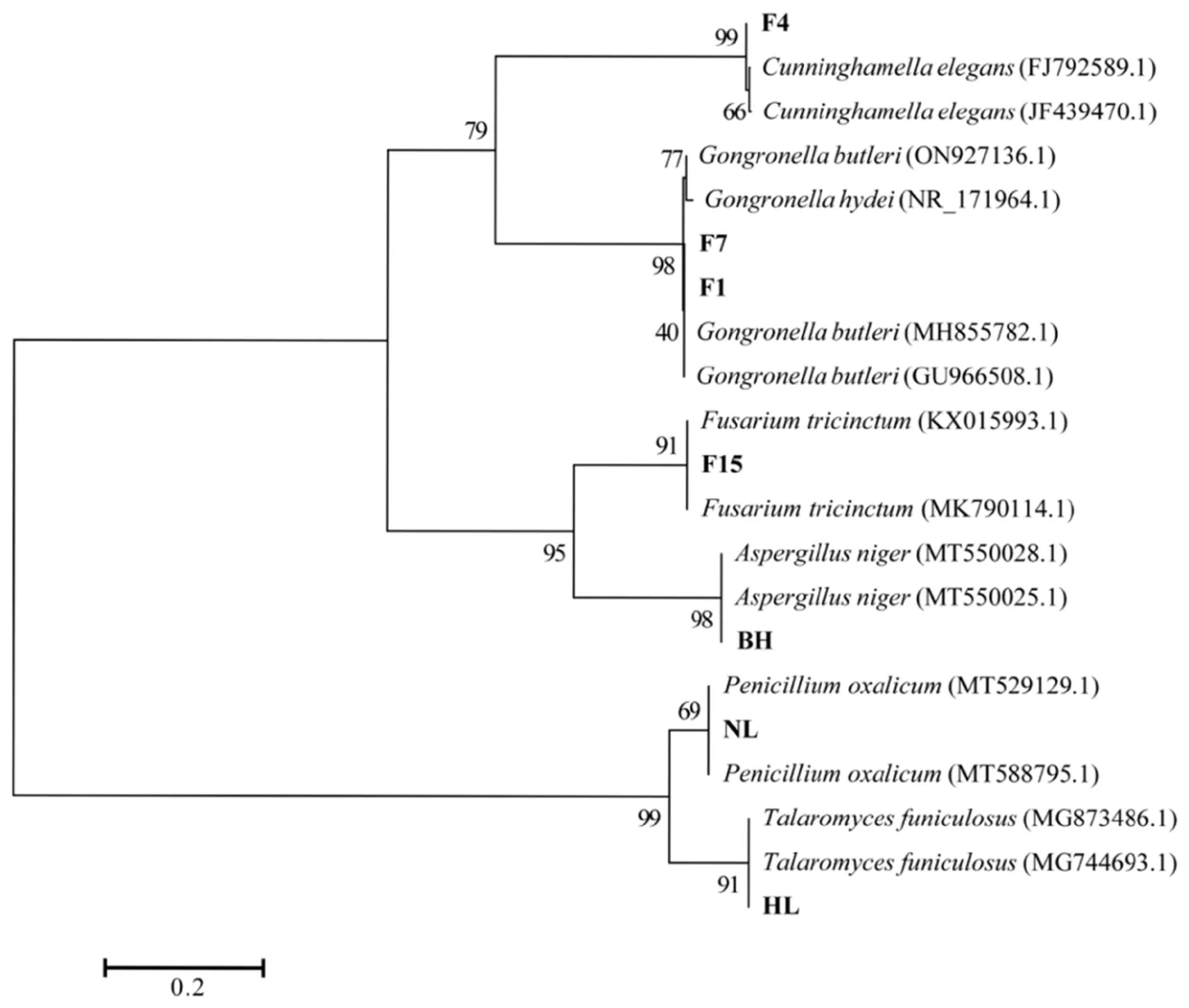
Phylogenetic tree of the seven fungal isolates constructed using the Neighbor-Joining method. F1 (*Gongronella butleri*), F4 (*Cunninghamella elegans*), F7 (*Gongronella hydei*), BH (*Aspergillus niger*), NL (*Penicillium oxalicum*), HL (*Talaromyces funiculosus*), and F15 (*Fusarium tricinctum*).

Representative ITS sequences with high similarity were downloaded from the GenBank database and aligned using Clustal W. A phylogenetic tree was then constructed using the Neighbor-Joining (NJ) method in MEGA 6.0 (Figure 1).

### 3.2. Effects of Fungi on the Growth and Physiological Indicators of Q. variabilis Seedlings

Inoculation with the seven fungal isolates significantly affected the aboveground growth of *Q. variabilis* seedlings (Table 1).

For aboveground fresh weight, isolate F15 showed the lowest value (2.95 g) and was significantly lower than CK (group a vs. b), representing a 22.98% reduction. Although F1, F4, F7, BH, and NL exhibited numerically lower aboveground fresh weights than CK, they belonged to the intermediate statistical group (ab) and therefore did not differ significantly from the control.

A similar pattern was observed for aboveground dry weight, where F15 (1.39 g, a) and F7 (1.40 g, a) showed significant reductions compared with CK (1.96 g), corresponding to decreases of 29.08% and 28.57%, respectively. Other isolates showed no significant differences or intermediate responses.

For aboveground water content, only minor variation was observed among treatments, ranging from 0.53% to 0.56%, with no strong statistical separation among most treatments.

In contrast, root fresh weight and root dry weight showed no significant differences among all treatments.

For root water content, isolates BH, NL, HL, and F15 showed lower values than CK, although the magnitude of reduction varied among isolates.

Overall, isolate F15 showed the strongest inhibitory effect on aboveground biomass, while root-related traits were less affected.

### 3.3. AMF Colonization in Quercus variabilis Roots

**Figure 2:**
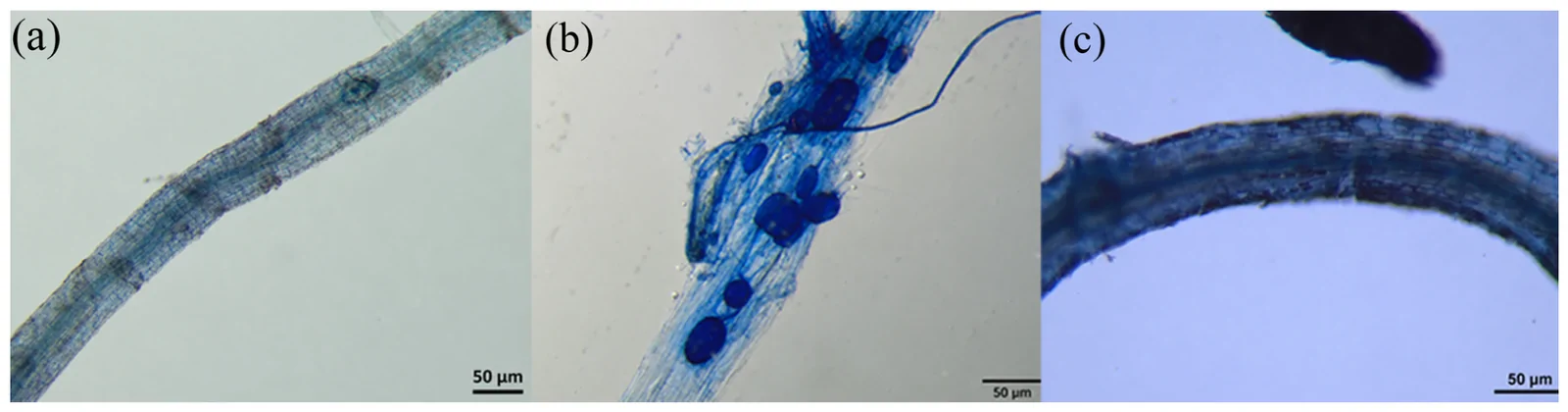
Microscopic observation of AMF colonization in *Quercus variabilis* roots. (**a**) CK treatment showing no AMF colonization structures. (**b**) AMF treatment showing typical vesicles and intraradical hyphae. (**c**) AMF + F15 treatment showing successful AMF colonization under pathogen stress. Scale bars = 50 μm.

Microscopic observations confirmed successful AMF colonization in the roots of *Quercus variabilis* seedlings (Figure 2). Typical AMF structures, including vesicles and intraradical hyphae, were observed in both the AMF and AMF + F15 treatments, whereas no typical AMF colonization structures were detected in the CK treatment. The average colonization rates of the AMF and AMF + F15 treatments were 53.89% and 45.73%, respectively, confirming that AMF successfully colonized the roots of *Q. variabilis* seedlings.

### 3.4. Effects of Different Treatments on Q. variabilis Seedling Growth, Physiology, and Substrate Nutrients

Significant differences were observed in the growth parameters of *Q. variabilis* seedlings among the different treatments (*p* < 0.05, Table 2). Overall, the AMF treatment showed better growth performance than the CK, AMF + F15, and F15 treatments. Plant height, ground diameter, and root length differed significantly among treatments. Compared with the F15 treatment, plant height in the AMF + F15 treatment increased by 12.79%, while AMF inoculation increased ground diameter by 28.57% and root length by 4.94% compared with F15 and CK, respectively.

Significant differences were also observed in the fresh and dry weights of the aboveground and root parts (*p* < 0.05, Table 3). Compared with F15, AMF inoculation increased aboveground fresh weight by 117.07% and root fresh weight by 352.26%. In the AMF + F15 treatment, aboveground dry weight and root dry weight increased by 70.71% and 102.00%, respectively, compared with F15. These results indicate that AMF inoculation promoted both vegetative growth and biomass accumulation of *Q. variabilis* seedlings.

Physiological indicators further showed differences among treatments (Table 4). POD and CAT activities in the AMF treatment were significantly higher than those in the F15 treatment (*p* < 0.05), whereas chlorophyll content did not differ significantly among treatments (*p* > 0.05). POD activity in the AMF treatment increased by 371.49% compared with F15, while CAT activity in the AMF + F15 treatment increased by 282.76% compared with F15. These results suggest that AMF inoculation enhanced the antioxidant enzyme activities of *Q. variabilis* seedlings, whereas chlorophyll content showed no significant response.

The nutrient contents and pH of the substrate also differed among treatments. Compared with CK, total nitrogen content decreased by 62.3% and 71.0% in the AMF and AMF + F15 treatments, respectively, and decreased by 67.9% and 75.3%, respectively, compared with F15. Similarly, total phosphorus content decreased by 40.0% and 20.0% in the AMF and AMF + F15 treatments, respectively, relative to CK, and by 78.6% and 71.4%, respectively, relative to F15. In contrast, total potassium content increased substantially in the AMF-treated groups, with increases of 94.7% and 105.3% in the AMF and AMF + F15 treatments, respectively, compared with CK, and increases of 56.4% and 65.0% compared with F15. Substrate pH also increased in the AMF-treated groups, by 2.0% and 5.9% in the AMF and AMF + F15 treatments, respectively, compared with CK, and by 5.2% and 9.1% compared with F15. These results indicate that AMF inoculation was associated with changes in substrate nutrient status and pH, particularly the depletion of total nitrogen and phosphorus and the increase in total potassium.

Overall, AMF inoculation improved seedling growth and biomass accumulation and enhanced antioxidant enzyme activities, accompanied by substantial changes in substrate nutrient status and pH.

### 3.5. Effects of Different Treatments on Rhizosphere Microbial Diversity and Community Composition of Q. variabilis Seedlings

Significant differences were observed in rhizosphere bacterial and fungal α-diversity among treatments (*p* < 0.05, Table 5). For bacterial communities, the ACE index was highest in the AMF treatment, followed by AMF + F15, CK, and F15, indicating higher bacterial richness in the AMF treatment. The Shannon index was highest in AMF + F15, followed by AMF, F15, and CK, whereas the Simpson index also differed significantly among treatments. For fungal communities, the ACE index followed the order CK > AMF > AMF + F15 > F15, while the Shannon index followed the order CK > AMF + F15 > AMF > F15. The Simpson index was highest in F15, followed by AMF + F15, AMF, and CK. These results indicate that AMF inoculation had different effects on bacterial and fungal α-diversity.

**Figure 3:**
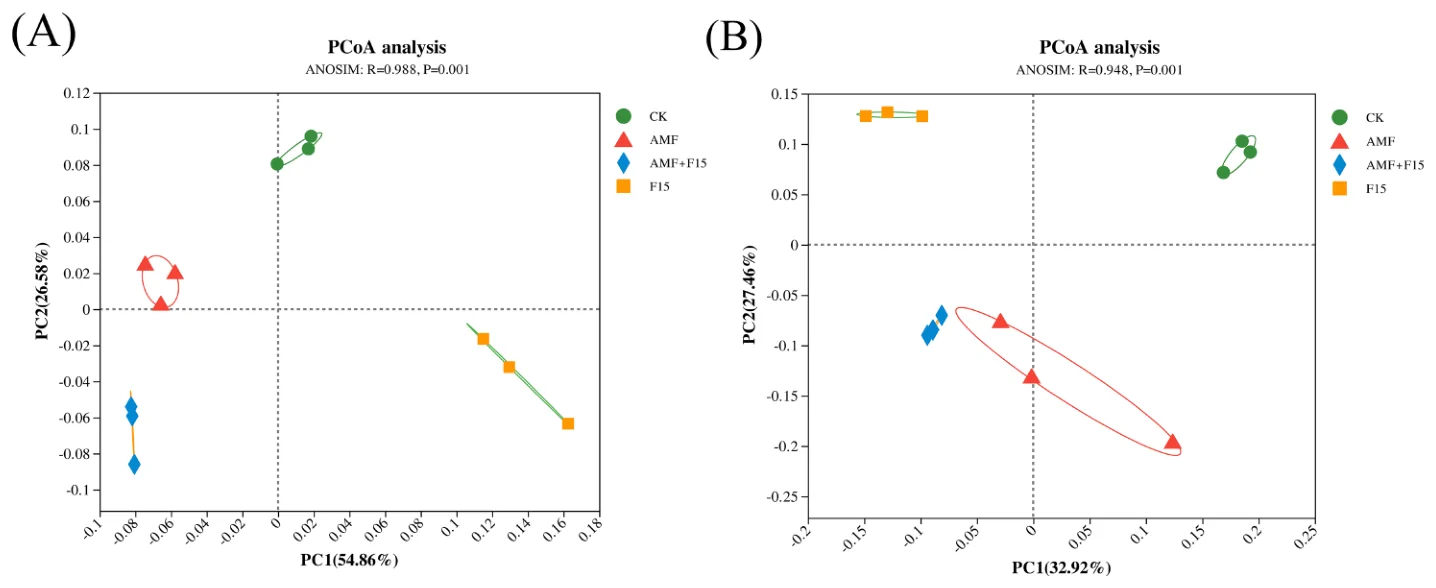
PCoA analysis of rhizospheric bacterial (**A**) and fungal (**B**) microbial communities in *Q. variabilis* seedlings under four treatments based on Bray–Curtis distances. ANOSIM R and *p* values are shown on the plots.

β-diversity analysis further revealed clear differences in rhizosphere microbial community structure among treatments (Figure 3). For bacterial communities, PCoA1 and PCoA2 explained 54.86% and 26.58% of the variation, respectively (Figure 3A), and ANOSIM indicated significant differences in bacterial community composition among treatments (R = 0.988, *p* = 0.001). The AMF and AMF + F15 groups occupied relatively similar positions in the PCoA ordination, whereas the other treatments showed greater separation. For fungal communities, PCoA1 and PCoA2 explained 32.92% and 27.46% of the variation, respectively (Figure 3B), and ANOSIM also indicated significant differences in fungal community composition among treatments (R = 0.948, *p* = 0.001). The AMF and AMF + F15 groups occupied relatively similar positions in the PCoA ordination, whereas the other treatments showed greater separation. These results indicate that AMF inoculation was associated with distinct shifts in both bacterial and fungal community structure.

**Figure 4:**
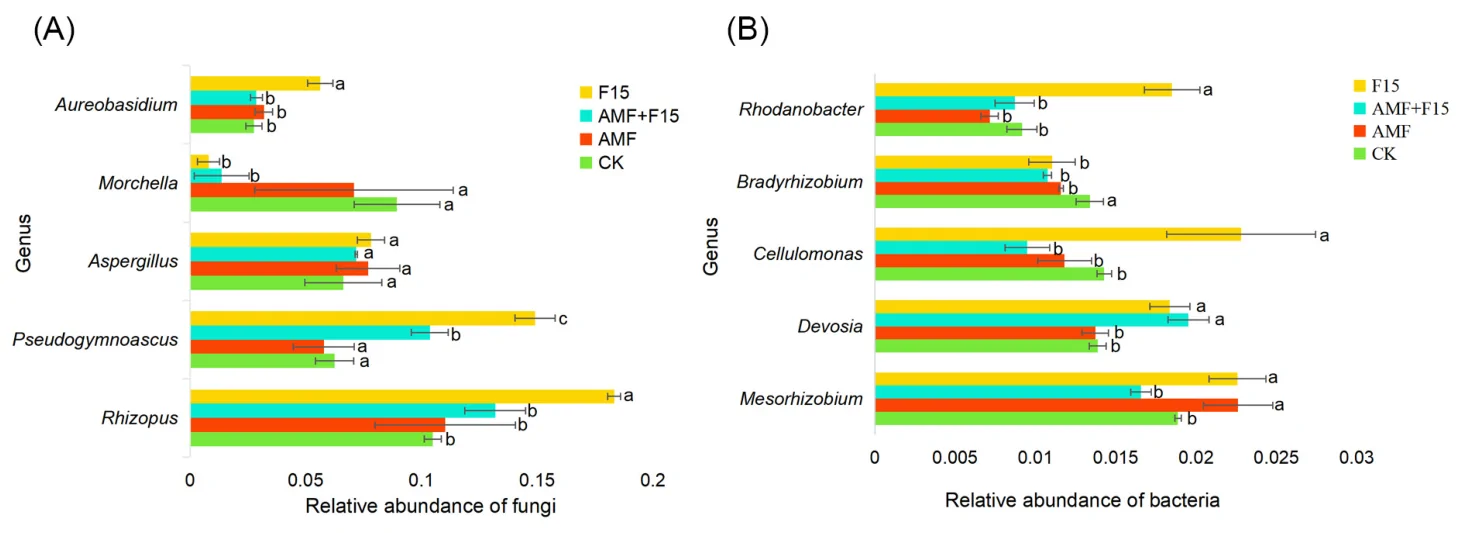
Compositional divergence of rhizospheric microbial communities in *Q. variabilis* seedlings across four treatments. Note: (**A**) fungi and (**B**) bacteria specifically depict the top 20 discriminative taxa with statistically significant abundance variations (*p* < 0.05). Different lowercase letters above the bars indicate significant differences among treatments (*p* < 0.05). F15 mentioned in the figures refers to *Fusarium tricinctum* F15. AMF refers to arbuscular mycorrhizal fungi.

Analysis of rhizosphere microbial community composition further identified specific taxa that differed among treatments (Figure 4). At the bacterial genus level, five of the top 20 abundant genera, including Cellulomonas, Mesorhizobium, Bradyrhizobium, Rhodanobacter, and Devosia, showed significant differences among treatments (*p* < 0.05). The relative abundance of Mesorhizobium was significantly higher in the AMF treatment than in the other groups. For fungal communities, the relative abundances of Rhizopus, Pseudogymnoascus, Aspergillus, and Aureobasidium were significantly higher in the F15 treatment than in the CK and AMF-treated groups, whereas Morchella was significantly more abundant in the AMF-treated groups than in the F15 treatment. These results demonstrate that AMF inoculation was associated with changes in the composition of both bacterial and fungal communities in the rhizosphere.

### 3.6. Functional Analysis of the Rhizosphere Microbial Community of Q. variabilis Seedlings Between Different Treatments

**Figure 5:**
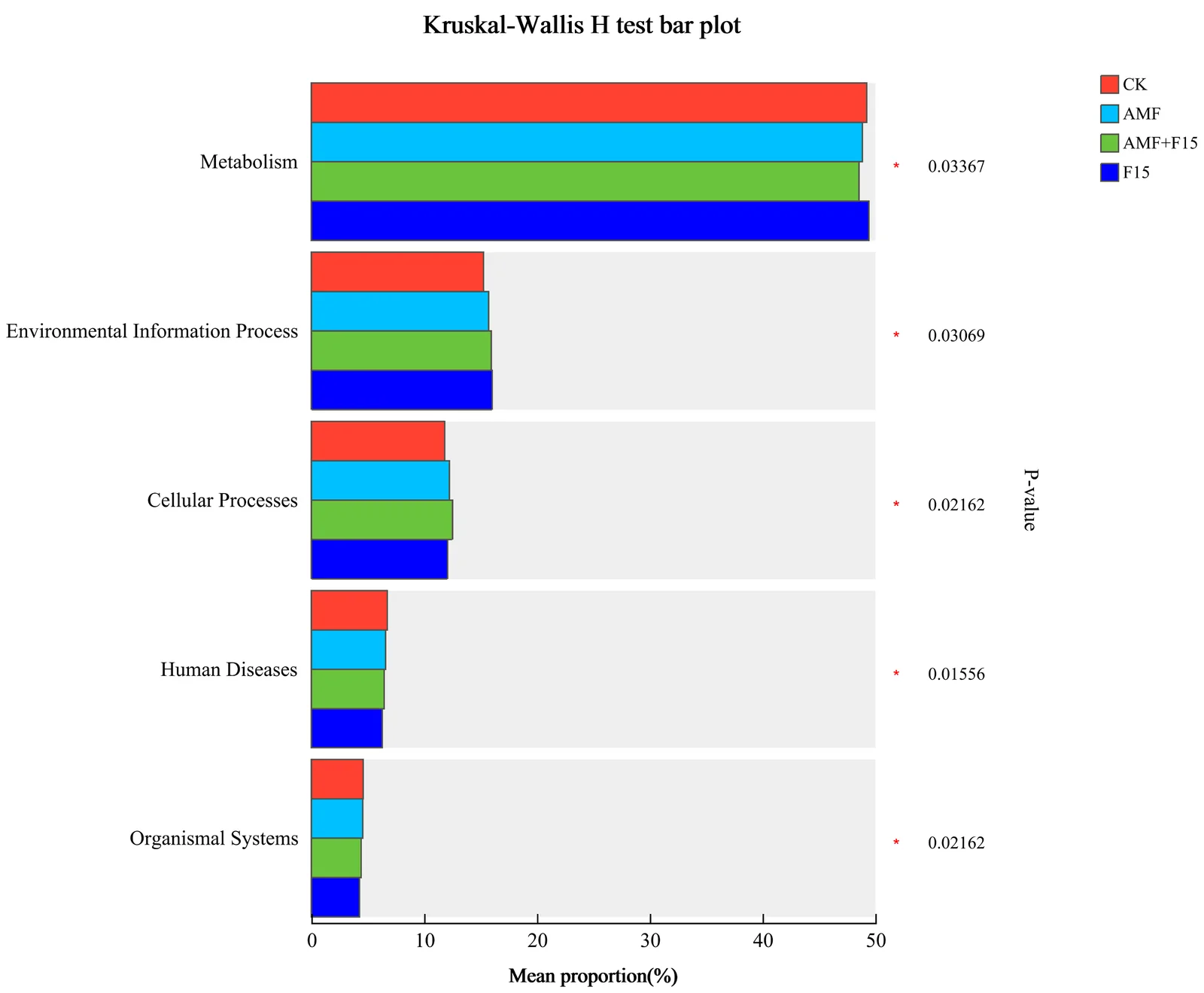
Functional analysis of the rhizosphere microbial community of *Q. variabilis* seedlings under different treatments. The asterisk (*) indicates a statistically significant difference (*p* < 0.05).

Figure 5 shows the functional differences among rhizosphere microorganisms in *Q. variabilis* seedlings under different treatments. Functional profiles were classified according to the KEGG Level 1 hierarchy, and the relative abundance of each functional category was compared among treatments. Five KEGG Level 1 functional categories showed significant differences among treatments (*p* < 0.05): Metabolism, Environmental Information Processing, Cellular Processes, Human Diseases, and Organismal Systems. Overall, the AMF-treated groups (AMF and AMF + F15) showed relatively higher proportions of genes assigned to Environmental Information Processing, Cellular Processes, and Organismal Systems, whereas the F15 treatment showed relatively higher proportions of genes assigned to Metabolism. These functional differences indicate that AMF inoculation and F15 challenge were associated with distinct shifts in the functional potential of the rhizosphere microbial community, providing functional-level evidence for the microbial community changes observed among treatments.

### 3.7. Correlation Analysis Between Environmental Factors and Microbial Communities

**Figure 6:**
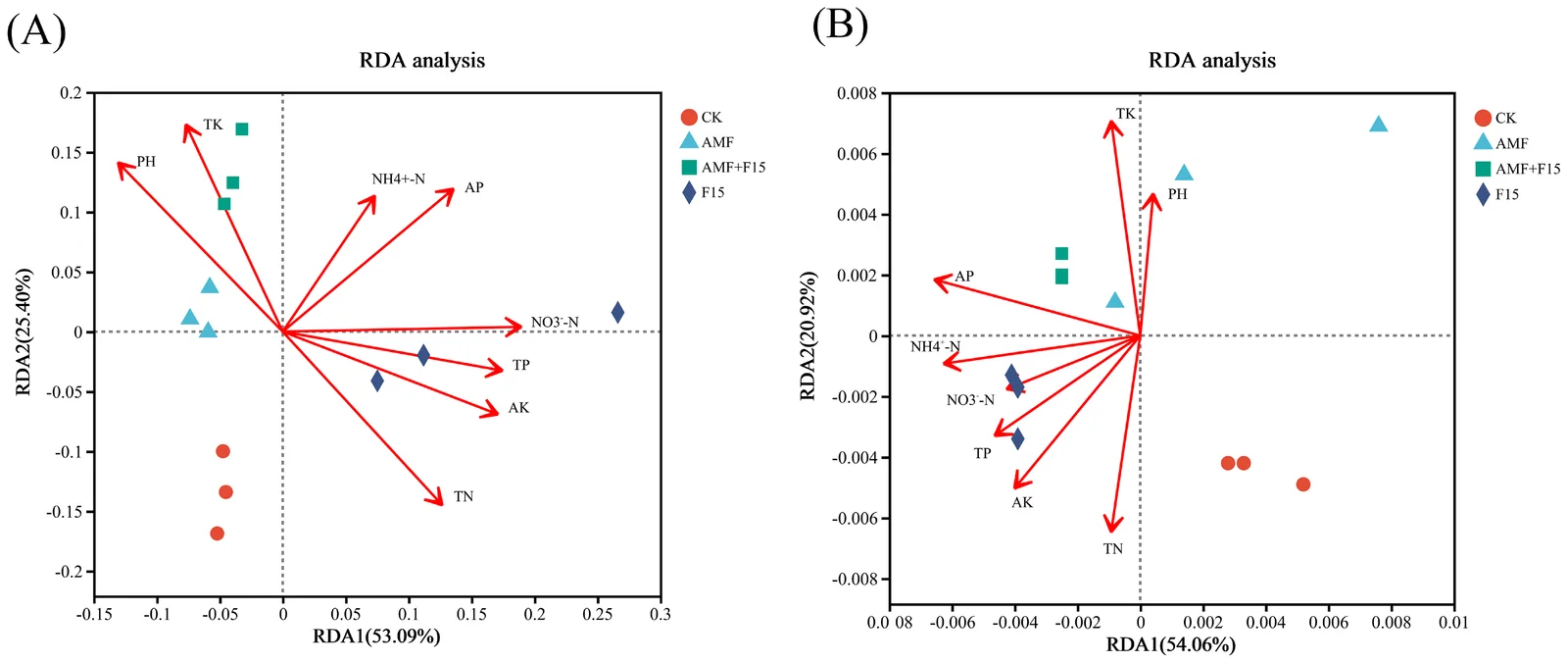
Redundancy analysis (RDA) illustrating the relationships between rhizospheric microbial communities and environmental factors under four treatments. (**A**) RDA of bacterial communities; (**B**) RDA of fungal communities. The arrows indicate environmental variables and their correlations with microbial community composition.

CANOCO5.0 was used to analyze the microbial community composition across different sampling points and sample types, in order to examine the relationship between rhizosphere microbial communities and physicochemical properties of *Q. variabilis* seedlings. Redundancy analysis (RDA) was performed to assess the influence of environmental factors on microbial distribution (Figure 6). The first two axes collectively explained 78.49% of the bacterial community variance (Figure 6A) and 74.98% of the fungal community variance (Figure 6B).

The bacterial community in the AMF treatment group was influenced mainly by pH and total potassium, while the fungal community was affected by pH, total potassium, and available phosphorus. In contrast, samples exposed to potential pathogens showed that both bacterial and fungal communities were associated with total nitrogen, total phosphorus, available potassium, and nitrate nitrogen. These trends were not observed in the control (CK) group.

## 4. Discussion

### 4.1. Fungal Pathogenicity Screening and Establishment of AMF Colonization

The fungal isolation and pathogenicity screening provided the basis for establishing the AMF–fungal stress system used in this study. Seven representative fungal isolates obtained from the rhizosphere of *Q. variabilis* showed different effects on seedling growth, with F15 exhibiting the strongest inhibitory effect, particularly on aboveground biomass. ITS sequencing identified the seven isolates as *Gongronella butleri* (F1), *Cunninghamella elegans* (F4), *Gongronella hydei* (F7), *Aspergillus niger* (BH), *Penicillium oxalicum* (NL), *Talaromyces funiculosus* (HL), and *Fusarium tricinctum* (F15). The internal transcribed spacer (ITS) region is widely used for fungal identification because it provides a practical molecular marker for distinguishing fungal taxa [34]. The combination of molecular identification and inoculation assays allowed the taxonomic identities of the isolates to be established while their effects on seedling growth were evaluated experimentally. The differential effects among the fungal isolates indicate that fungi associated with the *Q. variabilis* rhizosphere can exert distinct effects on their host, and that the growth response of the seedlings was not determined simply by the presence of a culturable rhizosphere fungus. The selection of F15 based on its relatively strong growth inhibition provided a biologically relevant fungal stress treatment for evaluating whether AMF could alleviate pathogen-associated growth suppression.

AMF colonization was successfully established in both the AMF and AMF + F15 treatments, with mean colonization rates of 53.89% and 45.73%, respectively. Microscopic observations of intraradical hyphae and vesicles further confirmed the establishment of the AMF symbiosis. Importantly, the presence of F15 did not prevent the establishment of AMF colonization under the conditions tested, indicating that the AMF–host association could be maintained during fungal challenge. The mean colonization rate was lower in the AMF + F15 treatment than in the AMF treatment, indicating that AMF colonization was reduced under F15 challenge. Together, these results established the experimental framework in which the subsequent growth, physiological, and rhizosphere microbiome responses could be evaluated under AMF inoculation with or without F15 challenge.

### 4.2. AMF Improves Seedling Growth and Physiological Performance Under Fungal Stress

The contrasting growth responses among treatments indicate that AMF colonization substantially modified the response of *Q. variabilis* seedlings to F15 challenge. F15 treatment resulted in poorer seedling growth, whereas AMF inoculation promoted plant height, ground diameter, root development, and biomass accumulation, and these growth advantages were partly maintained in the AMF + F15 treatment. The response was particularly evident in biomass accumulation and root development, suggesting that the beneficial effect of AMF was not limited to aboveground growth but extended to the belowground component of the seedlings. This pattern is consistent with the well-established role of AMF in promoting plant growth and influencing host nutrient acquisition [35]. Because the roots represent the interface where AMF colonization, host nutrient acquisition, and interactions with soil microorganisms occur, the improvement in root development may be particularly important for maintaining overall seedling growth under fungal challenge. Thus, the growth-promoting effect of AMF observed in this study may reflect an improved capacity of the root system to sustain seedling development when exposed to F15.

The physiological responses provide further insight into how AMF-associated growth improvement may be maintained under fungal stress. POD and CAT activities were higher in AMF-inoculated seedlings than in F15-treated seedlings, whereas SOD, chlorophyll, and protein contents showed no significant differences among treatments. The selective enhancement of POD and CAT suggests that AMF inoculation was associated with an altered antioxidant defense status rather than a generalized increase in all measured physiological indicators [36,37]. Under fungal challenge, increased activities of antioxidant enzymes may help seedlings maintain cellular redox homeostasis and limit oxidative damage, thereby supporting continued growth. Although oxidative damage was not directly measured in this study, the simultaneous improvement in growth and enhancement of POD and CAT activities are consistent with the possibility that AMF contributed to a stronger physiological capacity to cope with F15-associated stress.

The changes in substrate nutrient status provide an additional dimension for interpreting the growth response. AMF inoculation was accompanied by changes in total N, total P, total K, and pH, indicating that establishment of the AMF–plant association was associated with a modified nutrient environment. Previous research has also shown that AMF can influence nutrient-related microbial communities in the rhizosphere and hyphosphere [38]. Importantly, the lower substrate N and P contents observed in AMF-treated groups occurred together with improved seedling growth rather than growth suppression. This pattern may reflect greater nutrient utilization during seedling development, although the present measurements cannot distinguish plant uptake from other processes such as microbial transformation or changes in nutrient pools. Similarly, the higher total K content under AMF inoculation suggests altered K dynamics in the substrate, but does not by itself establish enhanced K uptake by the seedlings. Therefore, the nutrient results are better interpreted as evidence of changes in the plant–substrate nutrient environment rather than as direct measurements of AMF-mediated nutrient transfer.

Taken together, the growth, physiological, and substrate responses suggest that the effect of AMF under F15 challenge was likely generated through coordinated changes rather than through a single physiological trait. Successful AMF colonization was accompanied by improved root and shoot development, enhanced activities of key antioxidant enzymes, and changes in the surrounding nutrient environment. These responses may have collectively increased the ability of *Q. variabilis* seedlings to maintain growth under fungal challenge. Nevertheless, the present study does not allow the individual contribution of nutrient acquisition, antioxidant protection, or other AMF-mediated processes to be separated. The subsequent changes in rhizosphere microbial communities therefore provide an important additional perspective for understanding how AMF-associated effects on the plant may be connected with changes in the surrounding soil environment.

### 4.3. AMF Reshapes Rhizosphere Microbial Diversity and Community Composition

The metagenomic results indicate that AMF inoculation substantially reshaped the rhizosphere microbial community of *Q. variabilis*, although the responses of bacterial and fungal communities were not identical. Changes in α-diversity were relatively limited compared with the differences observed in community composition, suggesting that AMF-associated microbial restructuring was characterized more by changes in the relative abundance and distribution of taxa than by a simple increase or decrease in overall microbial diversity. This distinction is important because changes in community composition can occur without pronounced changes in community-level diversity indices, particularly when dominant or treatment-responsive taxa are redistributed within an established microbial community.

The β-diversity analysis provided clearer evidence of this community restructuring. Both bacterial and fungal communities showed distinct separation among treatments, with the AMF and AMF + F15 groups occupying relatively similar positions in the ordination plots and differing from the non-AMF treatments. This pattern suggests that AMF inoculation exerted a stronger influence on the overall structure of the rhizosphere microbiome than F15 alone. Such community-level effects are consistent with the recognized role of mycorrhizal associations in shaping interactions between plants and their surrounding microbial communities [39,40]. More importantly, the relatively similar positions of the AMF and AMF + F15 groups in the ordination plots suggest that the microbial environment associated with AMF colonization remained relatively similar in the presence of F15 challenge. Thus, the response to F15 appeared to occur within an AMF-modified rhizosphere rather than independently of AMF status. This provides a possible ecological basis for the improved plant performance observed in the AMF + F15 treatment.

The changes in specific microbial taxa further support the restructuring of the rhizosphere community. Among the bacterial taxa, differences in genera such as Cellulomonas, Mesorhizobium, Bradyrhizobium, Rhodanobacter, and Devosia indicate that AMF inoculation was associated with changes in bacterial groups involved in diverse rhizosphere ecological processes. Changes in the abundance of particular microbial taxa can contribute to broader shifts in community structure and functioning, particularly when these taxa occupy important ecological positions within the microbiome [40]. In particular, the higher relative abundance of Mesorhizobium in the AMF treatment suggests that AMF colonization may have altered the rhizosphere conditions favoring particular bacterial populations. However, the present study does not directly demonstrate the functional contribution of these taxa to plant growth or responses to F15 challenge, and their enrichment should therefore be interpreted as an indicator of community restructuring rather than evidence of direct biological mediation.

The fungal community showed a similarly treatment-dependent restructuring, but with a distinct taxonomic pattern. Genera including Rhizopus, Pseudogymnoascus, Aspergillus, and Aureobasidium were relatively more abundant in the F15 treatment, whereas Morchella showed a higher relative abundance in AMF-associated treatments. The enrichment of particular fungal taxa under F15 challenge may reflect the altered rhizosphere conditions associated with fungal stress, while the different fungal composition in AMF-treated seedlings indicates that AMF colonization was accompanied by a distinct fungal community state. Nevertheless, because the present analysis was based on relative abundance, an increase in one taxon may also reflect changes in other community members rather than an absolute increase in its population.

Taken together, these results suggest that AMF reshaped the rhizosphere microbiome primarily through changes in community structure and taxonomic composition rather than through a uniform increase in microbial diversity. The fact that the AMF and AMF + F15 groups occupied relatively similar positions in the PCoA ordination despite the presence of F15 is particularly noteworthy, because it suggests that AMF colonization may have established a distinct rhizosphere microbial state that remained relatively stable under fungal challenge. Such community restructuring provides a potential ecological link between AMF colonization and the improved growth and physiological performance of *Q. variabilis* seedlings observed in this study and provides a basis for further considering the functional characteristics of these microbial communities.

### 4.4. Microbial Functional Profiles and Environmental Associations Reveal Treatment-Related Rhizosphere Changes

The changes in rhizosphere microbial community structure were accompanied by distinct differences in microbial functional profiles. Based on the KEGG Level 1 classification, five functional categories differed significantly among treatments, including Metabolism, Environmental Information Processing, Cellular Processes, Human Diseases, and Organismal Systems. The AMF and AMF + F15 treatments showed relatively higher proportions of genes assigned to Environmental Information Processing, Cellular Processes, and Organismal Systems, whereas the F15 treatment showed a relatively higher proportion of genes assigned to Metabolism. These differences suggest that AMF inoculation was associated with a functional shift in the rhizosphere microbial community, extending the effects observed at the taxonomic level. Changes in microbial community composition can be accompanied by changes in microbiome functioning, particularly when treatment-associated shifts involve ecologically important microbial taxa [41]. In particular, the higher representation of functional categories related to environmental information processing and cellular processes in AMF-associated treatments may reflect a microbial community with greater potential for responding to and interacting with changes in the rhizosphere environment. However, these results represent differences in predicted functional potential rather than direct measurements of microbial activity, and therefore should not be interpreted as evidence that these functions were actually enhanced in vivo.

The relationships between microbial communities and substrate physicochemical properties provided further insight into the environmental context underlying these treatment-associated microbial differences. The bacterial community in the AMF treatment was mainly associated with pH and total K, whereas the fungal community was more closely associated with pH, total K, and available P. In contrast, the microbial communities under F15 challenge were associated with total N, total P, available K, and nitrate N. The first two ordination axes explained 78.49% of the variation in the bacterial community and 74.98% of the variation in the fungal community, indicating that the measured physicochemical variables were strongly associated with the observed variation in microbial community structure.

The different environmental associations among treatments are particularly informative when considered together with the nutrient and microbial results described above. AMF-associated microbial communities were closely related to pH and K-related variables, whereas F15-associated communities showed stronger relationships with N- and P-related variables as well as available K and nitrate N. This pattern suggests that AMF inoculation and F15 challenge were accompanied by different rhizosphere physicochemical states, which were in turn associated with distinct bacterial and fungal community structures. Thus, the microbial differences observed among treatments were not independent of the surrounding soil environment but occurred together with changes in substrate properties.

Taken together, the functional and environmental analyses extend the interpretation of the microbial community changes observed in this study. AMF inoculation was associated with both taxonomic restructuring and differences in predicted microbial functional profiles, while the ordination analysis linked these community differences to specific physicochemical properties of the substrate. These results support a model in which AMF-associated changes in the rhizosphere involve coordinated shifts in microbial community structure, functional potential, and the surrounding physicochemical environment. Nevertheless, because both functional annotation and ordination analysis provide community-level associations, the present data do not establish whether changes in physicochemical properties caused the observed microbial shifts or resulted from microbial and plant processes. Further experiments are therefore required to determine the causal relationships among AMF colonization, rhizosphere properties, microbial functions, and plant performance.

### 4.5. Integrated Interpretation of AMF Effects Under F15 Challenge

Integrating the physiological, nutritional, and rhizosphere microbiome results, our findings support a coordinated role of AMF in maintaining the performance of *Q. variabilis* seedlings under F15 challenge. Successful AMF colonization was accompanied by improved seedling growth and root development, together with enhanced POD and CAT activities. At the same time, AMF inoculation was associated with changes in substrate nutrient status and pH and with a distinct rhizosphere microbial community structure. These microbial changes extended beyond taxonomic composition to differences in predicted functional profiles and their associations with substrate physicochemical properties. Rather than indicating a single protective pathway, the consistency among these responses suggests that AMF-associated effects occurred across multiple interconnected levels of the plant–soil–microbiome system.

At the plant level, the maintenance of growth and biomass accumulation under AMF + F15 treatment, together with the changes in antioxidant enzyme activities, indicates that AMF colonization was associated with a physiological state that was better maintained during fungal challenge. At the rhizosphere level, the separation of AMF-associated microbial communities from those of non-AMF treatments suggests that AMF also modified the microbial environment surrounding the roots. The relatively similar bacterial and fungal community structures between AMF and AMF + F15 treatments are particularly relevant in this context, because they suggest that the AMF-associated rhizosphere state was maintained even when F15 was present. Changes in microbial functional potential and the distinct relationships between microbial communities and substrate properties further indicate that this altered rhizosphere state was accompanied by functional and environmental differences.

**Figure 7:**
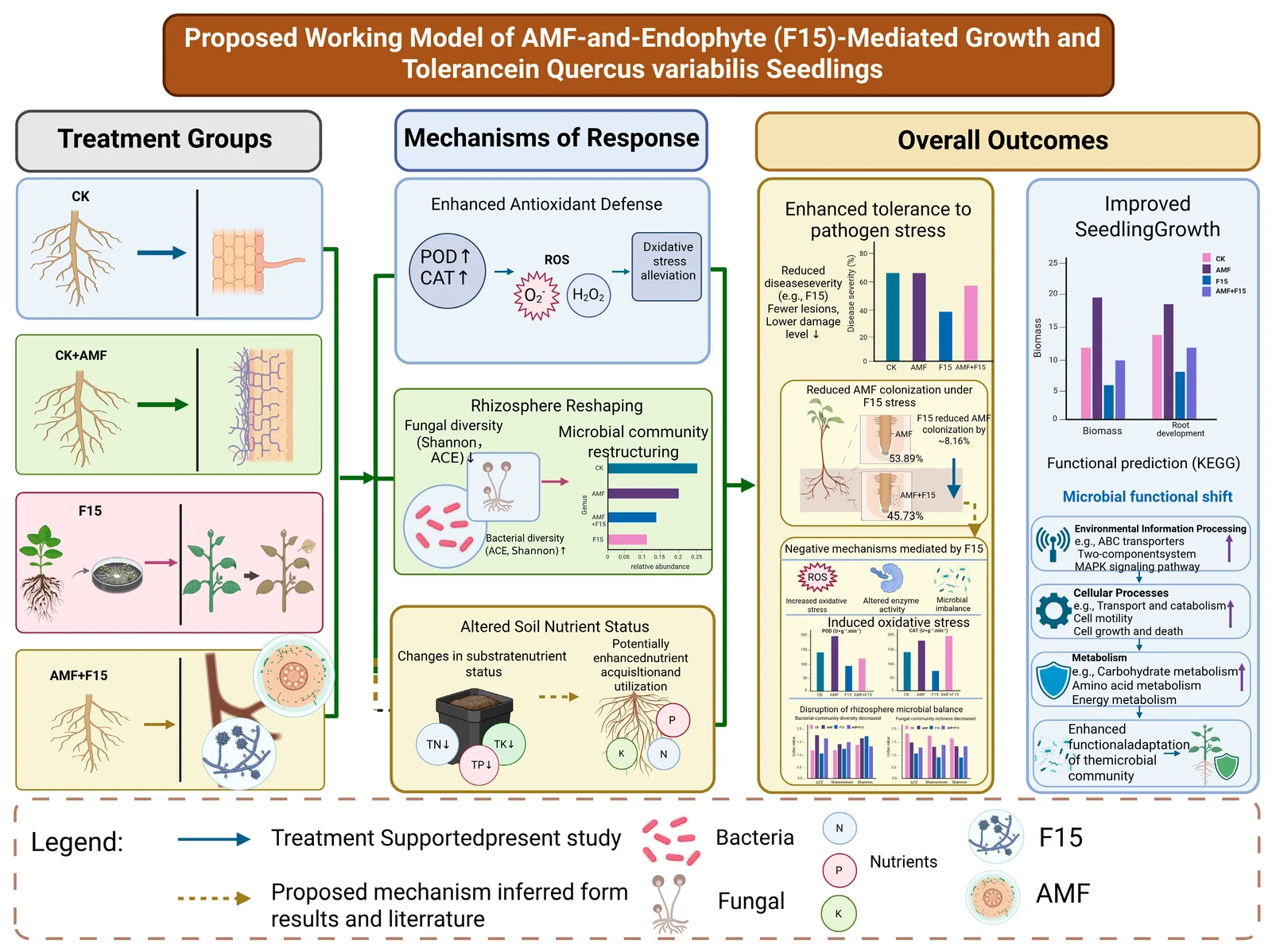
Proposed working model illustrating the potential effects of arbuscular mycorrhizal fungi (AMF) and fungal isolate F15 on seedling growth, antioxidant responses, rhizosphere microbial communities, and substrate nutrient dynamics in *Q. variabilis*. The model was developed by integrating the experimental findings of the present study with evidence from previous literature. Solid arrows indicate relationships directly supported by the experimental results, whereas dashed arrows represent proposed mechanisms inferred from the present findings and published studies.

These findings allow a working model to be proposed in which AMF colonization provides an initial symbiotic condition that is associated with improved root development and plant physiological performance, while simultaneously modifying the surrounding rhizosphere environment. As illustrated in Figure 7, this working model integrates the effects of AMF colonization on plant growth, physiological responses, substrate properties, and rhizosphere microbial communities under F15 challenge. The resulting changes in substrate properties and microbial community structure may further contribute to the establishment of a distinct rhizosphere microbial state under F15 challenge. In this framework, the effects of AMF on plant performance and rhizosphere microorganisms are not considered independent responses but are considered interconnected components of the plant–AMF–soil system. This interpretation is consistent with previous evidence that AMF symbiosis can influence plant responses to stress as well as interactions with surrounding soil microbial communities [42,43].

Importantly, the present study provides stronger evidence for coordinated associations than for direct causal relationships among these processes. We demonstrated AMF colonization, improved seedling growth, altered antioxidant enzyme activities, changes in substrate physicochemical properties, and restructuring of rhizosphere bacterial and fungal communities and their predicted functional profiles. However, the experiments did not directly determine whether the microbial community changes caused the improved plant performance, whether altered substrate properties drove microbial restructuring, or whether enhanced antioxidant activity was responsible for the greater growth of AMF-treated seedlings. Therefore, the proposed mechanism should be regarded as a working model that integrates the major patterns observed in this study rather than as a fully validated causal pathway. Further experiments involving targeted microbial isolation, functional validation, and direct measurements of nutrient uptake and oxidative damage will be required to resolve these relationships.

In addition, the soil physicochemical and metagenomic analyses were conducted with three biological replicates per treatment due to experimental cost considerations. This sample size represents a limitation of the present study, and the findings should be further validated with larger sample sizes in future studies.

## 5. Conclusions

This study demonstrates that AMF inoculation can improve the performance of *Q. variabilis* seedlings under F15 challenge. The beneficial effects of AMF were associated with coordinated changes in plant growth and physiological status, together with restructuring of the rhizosphere bacterial and fungal communities. These microbial changes were further accompanied by shifts in predicted functional profiles and their relationships with substrate physicochemical properties. Overall, our findings indicate that the effects of AMF under fungal challenge are associated with coordinated changes across the plant–AMF–rhizosphere microbiome system. This study provides new insight into the ecological role of AMF in supporting *Q. variabilis* seedling performance under fungal stress and provides a basis for further understanding plant–AMF–microbiome interactions.

## Figures and Tables

**Table 1:** The differences in growth indices of *Q. variabilis* seedlings with different fungi.

Treatments	Fresh Weight of Above-Ground Part (g)	Dry Weight of Above-Ground Part (g)	Water Content of the Above-Ground part (%)	Fresh Weight of the Root (g)	Dry Weight of the Root (g)	Water Content of the Root (%)
CK	3.83 ± 0.27 b	1.96 ± 0.50 b	0.56 ± 0.01 bc	1.69 ± 0.18 a	0.62 ± 0.11 a	0.67 ± 0.04 c
F1	3.57 ± 0.41 ab	1.71 ± 0.22 ab	0.55 ± 0.02 bc	1.51 ± 0.60 a	0.66 ± 0.23 a	0.64 ± 0.02 abc
F4	3.25 ± 0.28 ab	1.52 ± 0.17 ab	0.53 ± 0.02 ab	1.48 ± 0.24 a	0.48 ± 0.11 a	0.61 ± 0.03 ab
F7	3.21 ± 0.37 ab	1.40 ± 0.22 a	0.56 ± 0.02 c	1.32 ± 0.25 a	0.43 ± 0.05 a	0.66 ± 0.04 bc
F15	2.95 ± 0.41 a	1.39 ± 0.38 a	0.55 ± 0.03 bc	1.25 ± 0.35 a	0.42 ± 0.14 a	0.63 ± 0.05 abc
BH	3.37 ± 0.27 ab	1.72 ± 0.26 ab	0.54 ± 0.02 abc	1.39 ± 0.28 a	0.58 ± 0.08 a	0.60 ± 0.03 a
NL	3.40 ± 0.36 ab	1.86 ± 0.34 b	0.53 ± 0.01 a	1.45 ± 0.39 a	0.68 ± 0.27 a	0.60 ± 0.04 a
HL	3.89 ± 0.51 b	1.87 ± 0.20 b	0.55 ± 0.01 abc	1.18 ± 0.31 a	0.45 ± 0.13 a	0.62 ± 0.02 abc

F1 (*Gongronella butleri*), F4 (*Cunninghamella elegans*), F7 (*Gongronella hydei*), BH (*Aspergillus niger*), NL (*Penicillium oxalicum*), HL (*Talaromyces finiculosus*), F15 (*Fusarium tricinctum*). Note: *n* = 5; different lowercase letters indicate significant differences at *p* < 0.05.

**Table 2:** Rhizosphere substrate nutrient content of *Q. variabilis* seedlings under different treatments.

Process Name	CK	AMF	AMF + F15	F15
pH	6.99 ± 0.01 b	7.13 ± 0.02 c	7.40 ± 0.03 d	6.78 ± 0.02 a
Total nitrogen (%)	0.69 ± 0.01 b	0.26 ± 0.05 a	0.20 ± 0.01 a	0.81 ± 0.02 c
Total phosphorus (%)	0.05 ± 0.002 a	0.03 ± 0.002 a	0.04 ± 0.004 a	0.14 ± 0.03 b
Total potassium (%)	0.94 ± 0.02 a	1.83 ± 0.10 c	1.93 ± 0.10 c	1.17 ± 0.01 b
Available phosphorus (mg·kg^−1^)	23.03 ± 0.05 a	29.24 ± 0.22 b	32.97 ± 0.29 c	37.48 ± 0.48 d
Rapidly available potassium (mg·kg^−1^)	326.93 ± 5.24 c	115.38 ± 12.56 a	190.80 ± 2.26 b	639.28 ± 15.29 d
Nitrate nitrogen (mg·kg^−1^)	0.59 ± 0.15 a	1.94 ± 0.40 a	3.09 ± 0.15 a	44.50 ± 35.34 b
Ammonium nitrogen (mg·kg^−1^)	4.78 ± 1.45 b	1.98 ± 0.48 a	12.04 ± 0.48 d	9.25 ± 0.48 c

Note: *n* = 3; different lowercase letters indicate significant differences at *p* < 0.05. F15 mentioned in the table refers to Fusarium tricinctum F15. AMF refers to arbuscular mycorrhizal fungi.

**Table 3:** The differences in growth indices of *Q. variabilis* seedlings in different treatments.

Process Name	CK	AMF	AMF + F15	F15
Plant height (cm)	30.70 ± 2.40 b	33.00 ± 3.26 b	29.10 ± 2.04 ab	25.80 ± 1.68 a
Ground diameter (cm)	0.36 ± 0.15 a	0.45 ± 0.19 b	0.37 ± 0.02 a	0.35 ± 0.004 a
Root length (cm)	17.40 ± 2.41 bc	18.26 ± 2.05 c	14.70 ± 1.48 ab	14.10 ± 0.89 a
Above-ground part fresh weight (g)	4.83 ± 0.65 b	7.25 ± 0.55 c	6.03 ± 1.11 bc	3.34 ± 0.83 a
Above-ground dry weight (g)	2.65 ± 0.85 bc	3.45 ± 0.32 c	2.39 ± 0.56 ab	1.40 ± 0.35 a
Above-ground water content (%)	0.54 ± 0.02 a	0.54 ± 0.02 a	0.54 ± 0.01 a	0.50 ± 0.03 a
Fresh root weight (g)	4.97 ± 1.85 bc	7.01 ± 0.55 c	3.71 ± 1.13 b	1.55 ± 0.33 a
Root dry weight (g)	1.71 ± 0.67 bc	2.05 ± 0.45 c	1.01 ± 0.34 ab	0.50 ± 0.14 a
Root water content (%)	0.67 ± 0.05 a	0.71 ± 0.05 a	0.71 ± 0.05 a	0.67 ± 0.04 a

Note: *n* = 5; different lowercase letters indicate significant differences at *p* < 0.05. F15 mentioned in the tables refers to Fusarium tricinctum F15. AMF refers to arbuscular mycorrhizal fungi.

**Table 4:** The differences in physiological indices of *Q. variabilis* seedlings in different treatments.

Process Name	Chlorophyll Content (mg∙dm^−2^)	Protein Content (mg∙g^−1^)	POD (U∙g^−1^.min^−1^)	CAT (U∙g^−1^.min^−1^)	SOD (U∙g^−1^)
CK	3.86 ± 0.89 a	0.04 ± 0.001 a	3.68 ± 2.41 a	1.16 ± 0.34 a	81.53 ± 5.65 a
AMF	4.10 ± 0.10 a	0.05 ± 0.003 a	11.74 ± 4.48 b	2.19 ± 0.43 b	84.19 ± 3.58 a
AMF + F15	3.61 ± 0.02 a	0.04 ± 0.001 a	3.28 ± 1.46 a	2.22 ± 0.45 b	78.94 ± 4.53 a
F15	3.43 ± 0.63 a	0.05 ± 0.01 a	2.49 ± 0.86 a	0.58 ± 0.44 a	76.59 ± 2.27 a

Note: POD: peroxidase activity, CAT: catalase activity, and SOD: superoxide dismutase activity. *n* = 5; different lowercase letters indicate significant differences at *p* < 0.05. F15 mentioned in the table refers to Fusarium tricinctum F15. AMF refers to arbuscular mycorrhizal fungi.

**Table 5:** Differences in rhizosphere bacterial and fungal diversity in differently treated *Q. variabilis* seedlings.

Process Name	α-Diversity	CK	AMF	AMF + F15	F15
bacterial	ACE	3625.00 ± 23.00 a	3705.33 ± 18.01 b	3696.67 ± 11.02 b	3601.00 ± 7.00 a
	Shannon	5.03 ± 0.04 a	5.16 ± 0.03 b	5.23 ± 0.02 b	5.04 ± 0.07 a
	Simpson	0.02 ± 0.001 c	0.02 ± 0.001 b	0.01 ± 0.001 a	0.02 ± 0.001 b
	Shannoneven	0.61 ± 0.004 a	0.63 ± 0.004 bc	0.64 ± 0.002 c	0.62 ± 0.01 ab
	Simpsoneven	0.01 ± 0.001 a	0.02 ± 0.001 b	0.02 ± 0.001 c	0.02 ± 0.001 b
fungal	ACE	317.67 ± 18.15 c	283.67 ± 10.79 b	280.67 ± 4.04 b	248.67 ± 6.43 a
	Shannon	4.51 ± 0.21 b	4.24 ± 0.08 ab	4.31 ± 0.01 b	3.79 ± 0.03 a
	Simpson	0.03 ± 0.01 a	0.04 ± 0.004 a	0.04 ± 0.001 a	0.07 ± 0.003 b
	Shannoneven	0.78 ± 0.03 b	0.75 ± 0.02 b	0.77 ± 0.003 b	0.69 ± 0.01 a
	Simpsoneven	0.10 ± 0.02 b	0.09 ± 0.01 b	0.09 ± 0.003 b	0.06 ± 0.004 a

Note: ACE (abundance-based coverage estimator), Shannon diversity, Simpson dominance, Shannon evenness, and Simpson evenness. *n* = 3; different lowercase letters indicate significant differences at *p* < 0.05. F15 mentioned in the table refers to Fusarium tricinctum F15. AMF refers to arbuscular mycorrhizal fungi.

## Data Availability

The metagenomic sequencing data have been deposited in the NCBI Sequence Read Archive (SRA) under accession number PRJNA1088856. The ITS sequences obtained in this study are available in GenBank under accession numbers PZ413545–PZ413551. All other data supporting the findings of this study are available from the corresponding author upon reasonable request.

## Abbreviations

The following abbreviations are used in this manuscript:

AMF	arbuscular mycorrhizal fungi
*Q. variabilis*	*Quercus variabilis*
F1	*Gongronella butleri*
F4	*Cunninghamella elegan*
F7	*Gongronella hydei*
BH	*Aspergillus niger*
NL	*Penicillium oxalicum*
HL	*Talaromyces finiculosus*
F15	*Fusarium tricinctum*
